# Concomitant Gastric Acid Suppressants on the Survival of Patients with Non-Small-Cell Lung Cancer Treated with Epidermal Growth Factor Receptor Tyrosine Kinase Inhibitors: A Meta-Analysis

**DOI:** 10.1155/2022/3102641

**Published:** 2022-01-31

**Authors:** Jun Xia, Jiping Zhu, Lei Li, Shiqin Xu

**Affiliations:** ^1^Department of Respiratory Medicine, Jiangsu Province Hospital of Chinese Medicine, Affiliated Hospital of Nanjing University of Chinese Medicine, Nanjing 210029, China; ^2^Department of Anesthesiology, Women's Hospital of Nanjing Medical University, Nanjing Maternity and Child Health Care Hospital, Nanjing 210004, China

## Abstract

**Background:**

The influence of concomitant use of gastric acid suppressants (AS) on survival of patients with non-small-cell lung cancer (NSCLC) treated with epidermal growth factor receptor (EGFR) tyrosine kinase inhibitors (TKIs) is inconsistent according to previous studies. We performed a meta-analysis to evaluate the effect of additional AS in patients with NSCLC taking TKIs.

**Methods:**

Relevant observational studies were identified by a search of Medline, Embase, and Web of Science databases. Only studies with multivariate analyses were included. A random-effect model was used to combine the results.

**Results:**

Thirteen retrospective studies with 12259 patients were included. Pooled results showed that concomitant use of AS was associated with worse progression-free survival (PFS, adjusted hazard ratio (HR): 1.57, 95% confidence interval (CI): 1.31 to 1.89, *P* < 0.001; *I*^2^ = 65%) and overall survival (OS, adjusted HR: 1.38, 95% CI: 1.19 to 1.61, *P* < 0.001; *I*^2^ = 70%) in NSCLC patients taking TKIs. Sensitivity analysis limited to studies including NSCLC with EGFR mutation showed consistent results (HR for PFS: 1.53, *P*=0.003; HR for OS: 1.43, *P*=0.001). Subgroup analyses indicated that the association between concomitant use of AS and poor survival was not significantly affected by the category of AS used (proton pump inhibitors or histamine type-2 receptor antagonists) or the country of the study (Asian or non-Asian, *P* for subgroup analysis all >0.05).

**Conclusions:**

Concomitant use of AS in patients with NSCLC taking TKIs may be associated with poor survival outcomes.

## 1. Introduction

The epidermal growth factor receptor tyrosine kinase inhibitors (EGFR-TKIs) have become an effective treatment for patients with non-small-cell lung cancer (NSCLC) [1, 2]. For patients with NSCLC with EGFR mutations, TKIs have been reported to confer better survival benefits than conventional cytotoxic anticancer therapies [3]. Gastric acid suppressants (ASs), including proton pump inhibitors (PPIs) and histamine type-2 receptor antagonists (H2RAs), are frequently prescribed in NSCLC patients to alleviate symptoms of gastroesophageal diseases [4, 5]. It has been shown that approximately 30–50% of patients with lung cancer are using AS [6]. Since many TKIs are weak bases that exhibit pH-dependent solubility [7], coadministration of AS may reduce the absorption of TKIs by increasing the intragastric PH. Early studies showed that the plasma concentrations of gefitinib and erlotinib, two commonly used EGFR-TKIs, were significantly lower in NSCLC patients with concomitant AS compared to those without AS [8, 9], which raised the hypothesis that coadministration of EGFR-TKIs with AS may compromise the efficacy of TKIS in patients with NSCLC [10]. However, results of previous studies evaluating the association between concomitant AS on survival of patients with NSCLC taking EGFR-TKIs showed inconsistent results [11–23]. Some studies suggested that concomitant use of AS was associated with poor survival in these patients [11–13, 16–18, 20], while other studies did not show a significant association [14, 15, 19, 21–23]. Therefore, we performed a meta-analysis to comprehensively summarize current evidence regarding the influence of concomitant AS on the survival of patients with NSCLC taking EGFR-TKIs.

## 2. Methods

We followed the Meta-analysis of Observational Studies in Epidemiology (MOOSE) [24] and Cochrane's Handbook [25] guidelines during the design, performing, and presenting of the meta-analysis.

### 2.1. Search of Electronic Databases

We identified studies by a systematic search of Medline, Embase, and Web of Science electronic databases using the following terms: (1) “proton pump inhibitor” OR “proton pump inhibitors” OR “acid suppressive therapy” OR “antisecretory therapy” OR “PPI” OR “anti-ulcer agent” OR “antacid” OR “acid suppressants” OR “histamine type-2 receptor”; (2) “lung cancer”; and (3) “survival” OR “mortality” OR “prognosis” OR “death” OR “recurrence” OR “collapse.” Only clinical studies published in English were selected. An additional manual check-up for the reference lists of relevant original and review articles was performed as a supplement. The last literature search was conducted on June 10, 2021.

### 2.2. Selection of Eligible Studies

Inclusion criteria were (1) observational studies published as full-length articles; (2) included adult patients (18 years or above) with a confirmed diagnosis of NSCLC treated with EGFR-TKIs; (3) patients with concomitant use of AS, including PPIs and H2RAs, who considered as exposure; (4) compared progression-free survival (PFS) or overall survival (OS) between patients with and without concomitant use of AS; and (5) reported relative risk for the association between concomitant use of AS and survival outcomes in multivariate analysis including possible confounding factors. The definition of concomitant use of AS was consistent with the criteria adopted in the original articles. Reviews, noncohort studies, studies including non-NSCLC patients, studies with patients not using EGFR-TKIs, or studies that did not report PFS or OS were excluded.

### 2.3. Extraction of Data and Evaluation of Study Quality

Two of the authors independently conducted electronic database search, extraction of study data, and assessment of study quality according to the inclusion criteria described above. If there were discrepancies, they were resolved by discussion with the corresponding author. The extracted data included the following: (1) name of the first author, year of the publication, study design, and country of the study; (2) population characteristics, including diagnosis, total number, mean age, and sex of the patients; (3) TKI used; (4) definition of concomitant AS use and number of AS users in each study; and (5) outcomes reported and variables adjusted in the multivariate model analyzing the association between concomitant AS use and survival outcomes. The Newcastle–Ottawa Scale (NOS) [26] was used for study quality assessment, which included three domains such as defining of study groups, between-group comparability, and validation of the outcome. This scale totally scored from 1 to 9 stars, with 9 stars indicating the highest study quality level.

### 2.4. Statistical Methods

The primary objective of the study was to determine the association between concomitant AS use and survival outcomes in NSCLC patients taking TKIs. Hazard ratios (HRs) and 95% confidence intervals (CIs) were selected as the general outcome variable for the associations. Data of HRs and standard errors (SEs) were calculated from 95% CIs or *P* values, and an additional logarithmical transformation was performed to stabilize variance and normalize to the distribution [25]. Cochrane's *Q* test was used to evaluate the heterogeneity, and the *I*^2^ statistic was also estimated [27]. Heterogeneity was deemed to be significant if *I*^2^ > 50%. We used a random-effect model for data synthesis because this model has incorporated the potential between-study heterogeneity and could provide a more generalized result [25]. Sensitivity analyses were performed by omitting one individual study at a time to examine the robustness of the finding [28]. Moreover, sensitivity analysis limited to studies including patients with EGFR mutation was also performed. Influences of study characteristics on the association between concomitant AS and the survival outcomes were tested with predefined subgroup analyses, including categorizes of AS (PPIs or H2RAs), country of the study (Asian or non-Asian), and TKIs used. These variables were chosen for subgroup analyses because previous studies have suggested that differences in AS used, ethnicity of the patients, and categories of TKIs may influence the survival of survival in cancer patients receiving TKIs [29–31]. The funnel plots were constructed, and a visual inspection of the symmetry was conducted to reflect the publication bias. Begg's test and Egger's regression asymmetry test were further performed for the evaluation of potential publication bias [32, 33]. We used the RevMan (Version 5.1; Cochrane Collaboration, Oxford, UK) software for the statistical analyses.

## 3. Results

### 3.1. Results of Database Search

**Figure 1:**
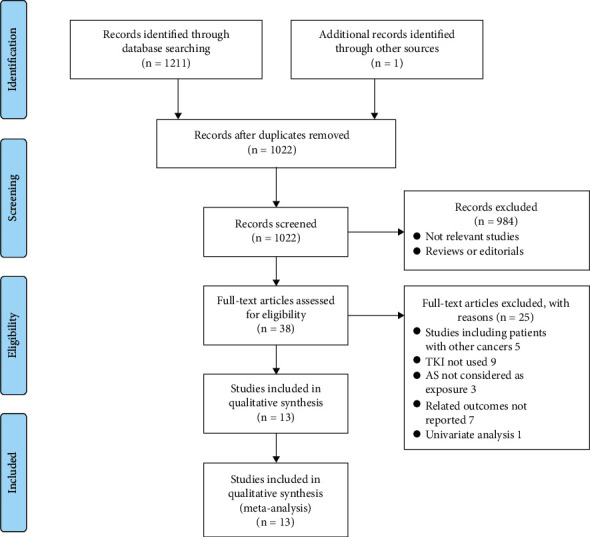
Flowchart of the database search and study identification.

The database search process is summarized in Figure 1. Briefly, 1212 articles were found in the initial literature search of the Medline, Embase, and Web of Science databases; after excluding the duplications, 1022 studies remained. An additional 984 were excluded through screening of the titles and abstracts mainly because of the irrelevance to the meta-analysis. The remaining 38 studies underwent a full-text review. Of the 38 studies, 25 were further excluded for the reasons listed in Figure 1. Finally, thirteen cohort studies [11–23] were included.

### 3.2. Characteristics of the Included Studies

As shown in Table 1, thirteen cohort studies [11–23] including 12259 patients with NSCLC that were treated with TKIs were included. Since one study reported two cohorts of patients taking dacomitinib or gefitinib, respectively [22], these two datasets were included independently in the meta-analysis. These studies were published between 2013 and 2021 and performed in Canada [11, 12], Spain [16], the United States [18, 22], Japan [15, 23], Singapore [14], and China [13, 17, 19–21], respectively. All of the studies were retrospective cohort studies and included patients with advanced NSCLC treated with TKIs. The mean ages of the patients varied between 61 and 76 years, and the proportions of males varied between 28 and 65%. In most of the included studies, the first-generation EGFR-TKIs such as erlotinib and gefitinib were used except for two studies, in which some of the patients also used the second-generation EGFR-TKIs such as afatinib and dacomitinib [21, 22]. Concomitant use of AS was validated by medical or prescription records in most of the studies except for one study, which was self-reported [11]. Concomitant use of PPIs or H2RAs was defined as exposure in most of the included studies except for four studies, which observed the concomitant use of PPIs [17, 18, 22] or H2RAs [23] only. Variables including age, sex, performance status, clinical stage, smoking history, comorbidities, and metastatic status were adjusted to a varying degree among the included studies. The NOS of the included studies was 7 to 9 stars, suggesting the generally good quality of the included studies (Table 2).

### 3.3. Association between Concomitant AS and PFS in NSCLC Patients Taking TKIs

**Figure 2:**
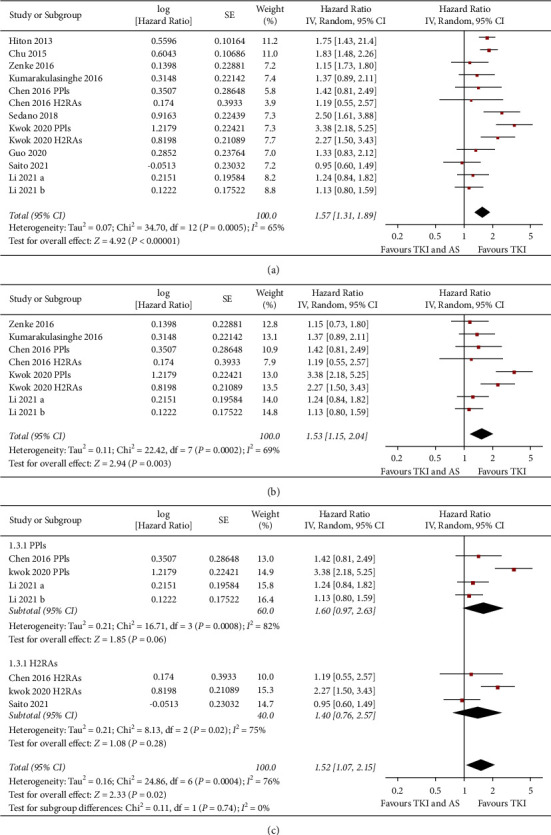
Forest plots for the meta-analysis of the association between concomitant AS use and PFS in NSCLC patients taking TKIs. (a) Forest plots for the overall meta-analysis, (b) forest plots for the sensitivity analysis in patients with EGFR mutation, and (c) forest plots for the subgroup analysis according to AS used.

**Figure 3:**
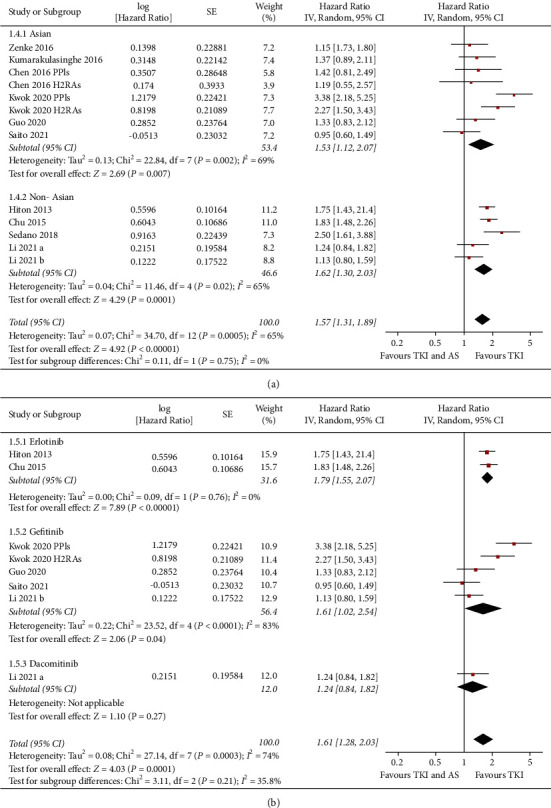
Forest plots for the subgroup analyses of the association between concomitant AS use and PFS in NSCLC patients taking TKIs. (a) Subgroup analysis according to the country of the study and (b) subgroup analysis according to TKIs used.

Ten studies reported the association between concomitant AS and PFS in NSCLC patients taking TKIs [11–16, 19, 20, 22, 23]. Pooled results with a random-effect model showed that concomitant AS was independently associated with a worse PFS in NSCLC patients taking TKIs (adjusted HR: 1.57, 95% CI: 1.31 to 1.89, *P* < 0.001; *I*^2^ = 65%; Figure 2(a)). Sensitivity analyses by excluding one study at a time showed consistent results (HR: 1.49 to 1.64, *P* all <0.05). Further sensitivity analyses limited to studies including patients with EGFR mutation also showed similar results (HR: 1.53, 95% CI: 1.15 to 2.04, *P*=0.003; *I*^2^ = 69%; Figure 2(b)). Subgroup analyses indicated that the association between concomitant use of AS and worse PFS was not significantly affected by category of AS used, country of the study, or category of TKIs (Figures 2(c), 3(a), and 3(b), *P* for subgroup difference all >0.05).

### 3.4. Association between Concomitant AS and OS in NSCLC Patients Taking TKIs

**Figure 4:**
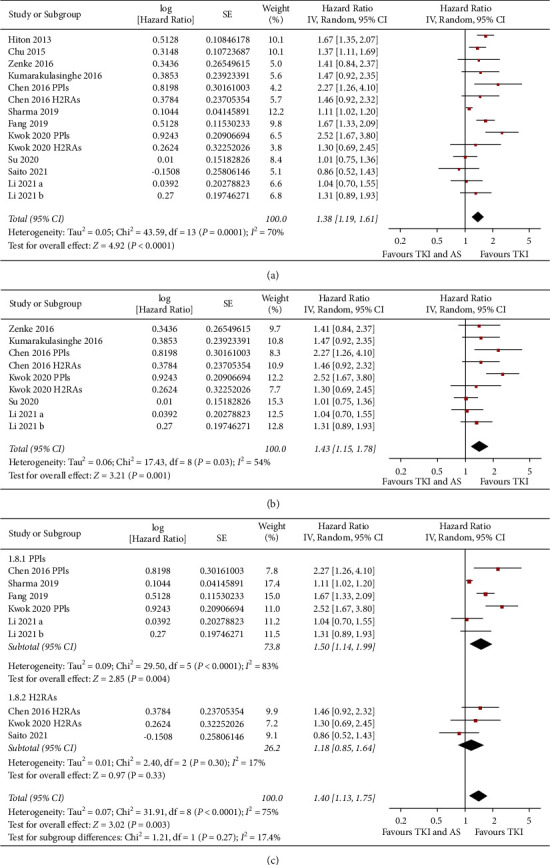
Forest plots for the meta-analysis of the association between concomitant AS use and OS in NSCLC patients taking TKIs. (a) Forest plots for the overall meta-analysis, (b) forest plots for the sensitivity analysis in patients with EGFR mutation, and (c) forest plots for the subgroup analysis according to AS used.

**Figure 5:**
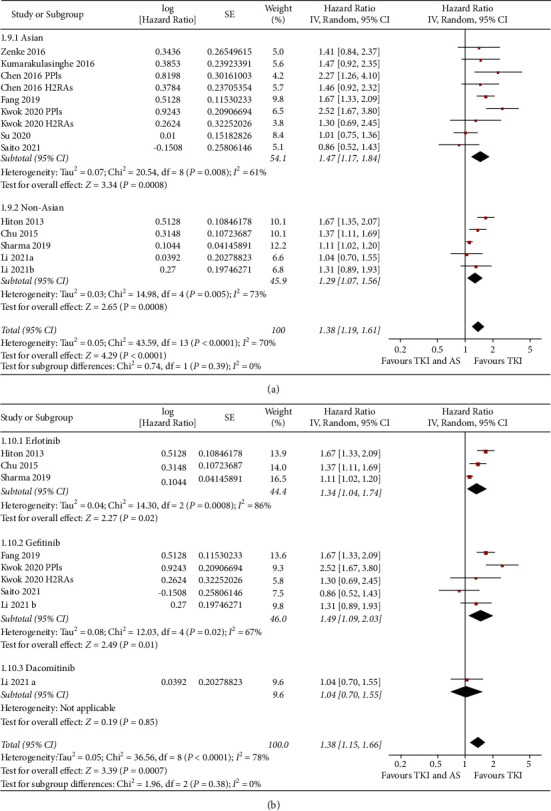
Forest plots for the subgroup analyses of the association between concomitant AS use and OS in NSCLC patients taking TKIs. (a) Subgroup analysis according to the country of the study and (b) subgroup analysis according to TKIs used.

Eleven studies reported the association between concomitant AS and OS in NSCLC patients taking TKIs [11–15, 17, 18, 20–23]. Results of meta-analysis showed that concomitant AS was independently associated with a worse OS (adjusted HR: 1.38, 95% CI: 1.19 to 1.61, *P* < 0.001; *I*^2^ = 70%; Figure 4(a)), which were consistent in sensitivity analyses by omitting one study at a time (HR: 1.33 to 1.43, *P* all <0.05) and limiting to studies including patients with EGFR mutation (HR: 1.43, 95% CI: 1.15 to 1.78, *P*=0.001; *I*^2^ = 54%; Figure 4(b)). Subgroup analyses also did not show a significant difference regarding the association between concomitant use of AS and OS according to the category of AS used, country of the study, or category of TKIs (Figures 4(c), 5(a), and 5(b), *P* for subgroup difference all >0.05).

### 3.5. Publication Bias

**Figure 6:**
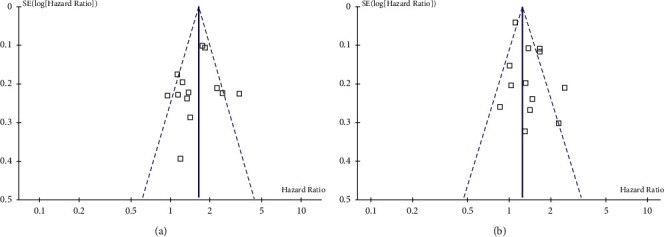
Funnel plots for the publication bias underlying the meta-analyses. (a) funnel plots for the meta-analysis of the association between concomitant AS use and PFS in NSCLC patients taking TKIs and (b) funnel plots for the meta-analysis of the association between concomitant AS use and OS in NSCLC patients taking TKIs.

Figures 6(a) and 6(b) show the funnel plots regarding the meta-analyses of the associations between concomitant AS with PFS and OS in NSCLC patients taking TKIs. The visual inspection found symmetry of the plots, which suggested a low risk of publication bias. Results of Begg's tests (*P*=0.34 and 0.77, respectively) and Egger's regression tests (*P*=0.29 and 0.47, respectively) also suggested the low risk of publication bias.

## 4. Discussion

In this meta-analysis, by pooling the results of available studies, we found that concomitant use of AS in NSCLC patients taking EGFR-TKIs was associated with worse PFS and OS as compared to those without AS. Further sensitivity analysis by excluding one dataset at a time and limiting to studies including patients with EGFR mutation only showed consistent results. Moreover, subgroup analysis did not show a significant different association in studies with PPIs or H2RAs, in Asian or non-Asian studies, or in studies with different TKIs. Taken together, these results suggested that concomitant use of AS may be independently associated with poor survival in NSCLC patients taking EGFR-TKIs. The combined use of AS and TKIs in patients with NSCLC should be cautious.

Several methodologic strengths of the meta-analysis should be noticed before the interpretation of the results. Firstly, an extensive search strategy was used to identify up-to-date studies relevant to the aim of the meta-analysis. This expanded search strategy was applied to avoid the missing of potentially relevant studies. In addition, only studies with multivariate analyses were included, aiming to provide an independent relationship between concomitant use of AS and poor survival of NSCLC patients taking TKIs. Finally, multiple predefined sensitivity and subgroup analyses were performed to evaluate the stability of the findings. Results of sensitivity analyses indicated that the possible independent relationship between concomitant use of AS and poor survival of NSCLC patients taking TKIs was not primarily driven by either of the included studies and remained significant in patients with EGFR mutations. Results of subgroup analysis showed that the above association was not significantly affected by categories of AS, location of the study, or TKIs used. As mentioned previously, the pharmacological basis for the finding is the potential drug interaction between AS and TKIs, which causes the reduced absorptions and plasma concentrations of TKIs in patients with NSCLC and compromised anticancer efficacies [34]. Considering the solid efficacy of EGFR-TKIs in NSCLC patients with EGFR mutations and the high prevalence of AS prescription in these patients, indications of AS should be strictly followed to reduce the unnecessary combined use of TKIs and AS in these patients.

We performed multiple subgroup analyses to influence whether the difference in categorizes of AS (PPIs or H2RAs), country of the study (Asian or non-Asian), and TKIs used may affect the influence of AS on survival in NSCLC patients treated with TKIs. Although subgroup analysis was usually used to analyze the source of heterogeneity, subgroup analyses could also be performed to investigate whether the outcomes are different according to the predefined subgroup variables (test for subgroup difference). Accordingly, for subgroups with *I*^2^ remains significant (>50%), it may indicate that differences in predefined subgroup analyses, such as the category of AS used, country of the study, or category of TKIs, were not the major source of heterogeneity. Although our subgroup analysis did not show that category of AS had significant influences on the association between AS and poor survival in NSCLC patients taking TKIs, the associations with poor PFS and OS were significant in the subgroup of studies with PPIs but nonsignificant in the subgroup of studies with H2RAs. Although validation in large-scale prospective cohort studies is needed, these findings might suggest a less influence of H2RAs than that of PPIs on the anticancer efficacy of TKIs. This may be explained by the fact that, compared to PPIs, H2RAs generally have a shorter duration of acid-suppressive effects and achieve a lower intragastric PH [35], which may influence less on the absorption of TKIs. Therefore, the use of HR2As may be considered over PPIs in NSCLC patients who need a combined treatment. Besides, results of subgroup analysis according to the type of TKIs showed that the association between concomitant AS and poor survival of NSCLC patients was significant in patients taking the first-generation TKIs including gefitinib and erlotinib but not significant in patients taking dacomitinib, the second-generation TKIs. These results should be interpreted with caution since only one dataset is available for dacomitinib, and the between-subgroup difference was not significant (*P*=0.21 for PFS and 0.38 for OS). However, the use of alternative TKIs that may be affected less by AS could be a resolution for NSCLC patients who have to use a combined treatment of AS and TKIs. For example, it has been shown that afatinib is highly soluble throughout the physiologic pH range of 1–7 and may therefore have fewer interactions with AS [36]. In addition, the plasma level of osimertinib, a third-generation TKIs, was not determined by coadministration with food or PPIs [37]. Future studies are warranted to determine the influence of concomitant AS use on the survival of NSCLC patients receiving these TKIs [38].

Our study has limitations, too. Firstly, all of the included studies were retrospective, which may expose the meta-analysis to a higher risk of recall and selection biases. Large-scale prospective cohort studies are needed to validate the findings. Besides, as previously mentioned, the results of the meta-analysis were primarily derived from studies with gefitinib and erlotinib. Future studies are needed to determine the influence of concomitant AS use on the survival of NSCLC patients receiving other EGFR-TKIs, such as afatinib, dacomitinib, and osimertinib. In addition, subgroup analyses should be interpreted with caution because of the limited datasets available for each subgroup. Moreover, influences of patient characteristics on the association between concomitant AS use and poor survival could not be fully analyzed in this study since it is a meta-analysis based on data from the study level. A meta-analysis based on individual patient data may be considered. In addition, for studies that were included in sensitivity analyses limited to patients with EGFR mutation [13–15, 19, 21, 22], only patients with EGFR mutations were included but not for patients without EGFR mutation. For other studies that did not specify the EGFR mutational status of the patients [11, 12, 16–19, 23], both patients with and without EGFR mutation were included. However, no subgroup data according to EGFR mutational status were provided in these studies. Accordingly, we could not perform subgroup analyses to compare the associations between patients with and without EGFR mutation. Future studies are warranted to determine whether the EGFR mutational status could affect the association between AS use and survival in NSCLC patients taking EGFR-TKIs. Finally, although we included only studies with multivariate analyses, there might be residual uncontrolled factors that may also confound the association, such as the dietary factors, other concurrent medications, and the time gap between administration of AS and TKIs.

## 5. Conclusions

In conclusion, results of this meta-analysis showed that current evidence based on retrospective studies suggested that concomitant use of AS may be independently associated with poor survival in NSCLC patients taking EGFR-TKIs such as gefitinib and erlotinib. The combined use of AS and TKIs in patients with NSCLC should be done with caution. Large-scale prospective cohort studies are needed to validate these findings and to clarify whether the type of AS and TKIs may affect the association.

## Figures and Tables

**Table 1:** Characteristics of the included studies.

Study	Country	Design	Patient characteristics	Sample size	Mean age (years)	Male (%)	TKI used	Definition of AS use	Number of AS users	Outcomes and main results reported: HR (95% CI)	Variables adjusted
Hilton 2013	Canada	R	Advanced or metastatic NSCLC after standard chemotherapy had failed	485	62	65	Erlotinib	Self-reported PPIs or H2RAs users at baseline or during follow-up	190	PFS: 1.75 (1.43∼2.13), OS: 1.67 (1.34∼2.05)	Age, sex, PS, weight loss, time from diagnosis, response to prior therapy, histologic subtype, race, HGB, LDH, and current smoking

Chu 2015	Canada	R	Stage IIIB/IV NSCLC patients	544	64	50	Erlotinib	PPIs or H2RAs user confirmed by the database of prescription medications	124	PFS: 1.83 (1.48∼2.25), OS: 1.37 (1.11∼1.69)	Age, sex, PS, and histologic subtype

Zenke 2016	Japan	R	Patients with advanced NSCLC with EGFR mutations	130	64	37	Erlotinib or gefitinib	Medical records approved users of PPIs or H2RAs	47	PFS: 1.15 (0.73∼1.79), OS: 1.41 (0.83∼2.35)	Age, sex, PS, smoking, prior therapy, and bone metastasis

Kumarakulasinghe 2016	Singapore	R	Patients with advanced NSCLC with EGFR mutations	157	62	53	Erlotinib or gefitinib	Medical records approved users of PPIs or H2RAs	55	PFS: 1.37 (0.89∼2.12), OS: 1.47 (0.92∼2.35)	Age, sex, race, PS, CCI, smoking, brain and liver metastasis

Chen 2016	China	R	Patients with advanced NSCLC with EGFR mutations	269	65	42	First-generation EGFR-TKIs	Medical records approved users of PPIs or H2RAs	57	For PPIs: PFS: 1.42 (0.81∼2.49), OS: 2.27 (1.26∼4.11); for H2RAs: PFS: 1.19 (0.55∼2.57), OS: 1.46 0.92∼2.33);	Age, sex, DM, smoking, PS, histologic type, and bone, liver, or pleura metastasis

Sedano 2018	Spain	R	Stage III/IV NSCLC patients	163	70	64	Erlotinib or gefitinib	Medical records approved users of PPIs or H2RAs	118	PFS: 2.5 (1.61∼3.88)	Age, sex, PS, and treatment line

Sharma 2019	US	R	Old NSCLC patients	7448	76	48	Erlotinib	PPIs user confirmed by the database of prescription medications	1616	OS: 1.11 (1.02∼1.20)	Age, sex, and comorbidities

Fang 2019	China	R	NSCLC patients	1278	NR	36	Gefitinib	PPIs user confirmed by the database of prescription medications	309	OS: 1.67 (1.33∼2.09)	Age, sex, comorbidities, and income

Kwok 2020	China	R	Advanced lung AC with EGFR mutations	193	68	28	Gefitinib	Medical records approved users of PPIs or H2RAs	61	For PPIs: PFS: 3.38 (2.18∼5.25), OS: 2.52 (1.67∼3.79); for H2RAs: PFS: 2.27 (1.54∼3.52), OS: 1.30 (0.74∼2.62);	Age, sex, smoking, primary EGFR mutation and presence of brain metastasis

Guo 2020	China	R	Stage IIIB/IV NSCLC patients	188	61	42	Gefitinib	Medical records approved users of PPIs or H2RAs	49	PFS: 1.33 (0.78∼1.98)	Age, sex, PS, EGFR mutation, cancer stage, and brain metastasis

Su 2020	China	R	Stage IIIB/IV NSCLC patients with EGFR mutations	853	66	36	Gefitinib, erlotinib, and afatinib	Medical records approved users of PPIs or H2RAs	92	OS: 1.01 (0.75∼1.36)	Age, sex, PS, smoking, clinical stage, previous therapy, CCI, and histologic type

Saito 2021	Japan	R	Stage IIIB/IV NSCLC patients	87	63	45	Gefitinib	Medical records approved users of H2RAs	31	PFS: 0.95 (0.60∼1.48), OS: 0.86 (0.52∼1.43)	Age, sex, PS, smoking, EGFR mutation, clinical stage, histologic type, previous chemotherapy, and liver dysfunction

Li 2021a^*∗*^	US	R	Patients with advanced NSCLC with EGFR-activating mutations	235	64	37	Dacomitinib	Medical records approved users of PPIs	83	PFS: 1.24 (0.84∼1.81), OS: 1.04 (0.70∼1.55)	Age, sex, race, baseline BW, PS, smoking, EGFR mutation type, number of target lesions, and organs with metastases

Li 2021b^*∗*^	US	R	Patients with advanced NSCLC with EGFR-activating mutations	229	63	42	Gefitinib	Medical records approved users of PPIs	70	PFS: 1.13 (0.80∼1.59), OS:1.31 (0.89∼1.93)	Age, sex, race, baseline BW, PS, smoking, EGFR mutation type, number of target lesions, and organs with metastases

^*∗*^The study by Li et al. (2021) included two cohorts using dacomitinib or gefitinib, respectively, and these two datasets were included independently. TKI, tyrosine kinase inhibitor; AS, acid suppressants; R, randomized; NSCLC, non-small-cell lung cancer; AC, adenocarcinoma; EGFR, epithelial growth factor receptor; PPIs, proton pump inhibitors; H2RAs, histamine type-2 receptor antagonists; PFS, progression-free survival; OS, overall survival; PS, performance status; HGB, hemoglobin; LDH, lactate dehydrogenase; CCI, Charlson Comorbidity Index; BW, body weight; DM, diabetes mellitus.

**Table 2:** Details of study quality evaluation via the Newcastle–Ottawa scale.

Study	Representativeness of the exposed cohort	Selection of the nonexposed cohort	Ascertainment of exposure	Outcome not present at baseline	Control for age	Control for other confounding factors	Assessment of outcome	Enough long follow-up duration	Adequacy of follow-up of cohorts	Total
Hilton 2013	1	1	0	1	1	1	1	1	1	8
Chu 2015	1	1	1	1	1	1	1	1	1	9
Zenke 2016	1	1	1	1	1	1	1	1	1	9
Kumarakulasinghe 2016	1	1	1	1	1	1	1	1	1	9
Chen 2016	1	1	1	1	1	1	1	1	1	9
Sedano 2018	1	1	1	1	1	1	1	1	1	9
Sharma 2019	0	1	1	1	1	0	1	1	1	7
Fang 2019	1	1	1	1	1	0	1	1	1	8
Kwok 2020	0	1	1	1	1	1	1	1	1	8
Guo 2020	1	1	1	1	1	1	1	1	1	9
Su 2020	1	1	1	1	1	1	1	1	1	9
Saito 2021	1	1	1	1	1	1	1	1	1	9
Li 2021	1	1	1	1	1	1	1	1	1	9

## Data Availability

The data used to support the findings of this study are available from the corresponding author upon request.

## Additional Points

*Review Criteria*. (i) A comprehensive search strategy using combined keywords was designed. (ii) Medline, Embase, and Web of Science were searched up to June 10, 2021. (iii) Studies evaluating the association between concomitant AS use and survival outcomes in NSCLC patients taking TKIs were included. *Message for the Clinic*. (i) Concomitant use of AS may be independently associated with poor survival in NSCLC patients taking EGFR-TKIs. (ii) Combined use of AS and TKIs in patients with NSCLC should be cautious.
